# High-flux hemodialysis as rescue therapy for high-dose methotrexate toxicity: case series and clinical insights

**DOI:** 10.3389/fmed.2025.1495705

**Published:** 2025-03-14

**Authors:** Ana Carolina Nakamura Tome, Karise Fernades Santos, Emerson Quintino Lima, Rodrigo Jose Ramalho

**Affiliations:** Hospital de Base, Faculty of Medicine of São José do Rio Preto - FAMERP, São José do Rio Preto, Brazil

**Keywords:** methotrexate toxicity, high-dose methotrexate, acute kidney injury, high-flux hemodialysis, extracorporeal treatment

## Abstract

High-dose methotrexate (HD-MTX) toxicity represents a significant clinical challenge in oncology, commonly resulting in acute kidney injury (AKI), myelosuppression, and potentially life-threatening multiorgan failure. This case series describes three patients treated at Hospital de Base in São José do Rio Preto, Brazil, who developed HD-MTX-induced AKI following the administration of chemotherapy. Two patients had hematologic malignancies and one had osteosarcoma. All received conventional rescue therapies, including leucovorin and aggressive hydration, but demonstrated persistent elevation of serum methotrexate levels, necessitating the initiation of high-flux hemodialysis (HF-HD). The mean number of HF-HD sessions required was 5.3 ± 2.5, and the mean relative reduction in serum methotrexate concentration was 44.5 ± 19.1%. These findings suggest that HF-HD is an effective therapeutic option for HD-MTX toxicity management in settings where glucarpidase is not readily available, although repeated sessions may be required due to the observed rebound in serum methotrexate levels.

## Introduction

Methotrexate (MTX), a folic acid analog, disrupts critical metabolic pathways by exhibiting a high affinity for dihydrofolate reductase (DHFR), thereby inhibiting its activity upon cellular uptake. This inhibition impairs the reduction of dihydrofolate to tetrahydrofolate, a cofactor indispensable for the biosynthesis of thymidine and purines, which are essential precursors for DNA synthesis. Consequently, MTX effectively suppresses cellular proliferation and protein synthesis, ultimately inducing cell death. This cytotoxic effect is particularly pronounced in rapidly dividing cells, notably those in the S-phase of the cell cycle, where the demand for DNA precursors is elevated. Thus, MTX is classified as an S-phase-specific cytotoxic agent (1).

MTX is extensively utilized in the treatment of hematological malignancies and solid tumors. Intravenous doses of 500 mg/m^2^ or higher are classified as high-dose methotrexate (HD-MTX) and are incorporated into therapeutic regimens for osteosarcoma, central nervous system (CNS) involvement in patients with leukemia and high-risk lymphoma, leptomeningeal metastases, and primary CNS lymphoma (1). MTX undergoes conversion to 7-hydroxymethotrexate by hepatic aldehyde oxidase and can subsequently be transformed into methotrexate polyglutamate (MTXPG) derivatives via the enzyme folylpolyglutamate synthase. This metabolic pathway produces a metabolite that enhances and prolongs MTX’s antiproliferative effects (2). However, this process also contributes to the increased severity and incidence of its associated adverse effects (3, 4).

The most critical adverse effect associated with MTX therapy is myelosuppression, which is responsible for most of the rare fatal outcomes. Additional side effects include mucositis, neurotoxicity, pneumonitis, and, in severe cases, multi-organ failure. Despite the use of antiemetics, vomiting occurs in 10–30% of patients. Transient, reversible hepatotoxicity may affect up to 60% of cases, while hyperbilirubinemia is observed in up to 25%. Renal involvement poses a significant challenge, both as a serious adverse effect and as a risk factor. The kidneys are responsible for excreting at least 90% of MTX, either in its unchanged form or as its metabolite (5). In cases of renal impairment, serum MTX levels may remain elevated and persist over prolonged periods (1, 2).

AKI has been reported in 2–12% of patients receiving HD-MTX (1, 6). The nephrotoxicity associated with HD-MTX is primarily attributed to the precipitation of MTX and its insoluble metabolites within the renal tubules, leading to acute tubular necrosis (7). Microscopic analysis of urinary sediment may reveal well-defined, brown crystals that are birefringent under polarized light. Renal biopsy findings can demonstrate MTX crystals with distinctive annular structures, a golden-brown hue in hematoxylin and eosin (H&E) staining, argyrophilia, and a radial pattern when examined under polarized light (8). Additionally, afferent arteriolar vasoconstriction, which reduces glomerular filtration rate, has also been described (1).

Recent studies have highlighted specific risk factors and genetic predispositions influencing MTX toxicity. Alves et al. investigated the administration of high-dose methotrexate in outpatient settings without drug monitoring and identified that age, body mass index and glomerular filtration rate (GFR) were significant risk factors for AKI. Among those patients with AKI, only 36% had their MTX serum level measured. Their findings underscore the importance of close monitoring to prevent adverse outcomes in vulnerable patient populations (9). Additionally, Pitakkitnukun et al. explored genetic polymorphisms and clinical parameters associated with renal toxicity in Thai patients receiving high-dose methotrexate, further elucidating the interplay between genetic and clinical factors in predicting adverse outcomes (10).

The standard treatment for HD-MTX-induced nephrotoxicity involves aggressive hydration, urinary alkalinization (pH >7.0), and early rescue with leucovorin (7). Glucarpidase (carboxypeptidase-G2, CPDG2), a recombinant bacterial enzyme that hydrolyzes MTX into its inactive metabolites [2,4-diamino-N10-methylpteroic acid (DAMPA) and glutamate], leading to an approximate 98% reduction in MTX concentrations, is regarded as the preferred therapeutic option for managing delayed MTX clearance, particularly in the context of elevated serum MTX levels and AKI. Although CPDG2 is highly effective and generally well-tolerated, its use is limited by immediate unavailability and its substantial cost (exceeding $100,000 to treat a 70 kg adult) (7). Recent studies have shown that lower doses of glucarpidase can be effective, thereby reducing the overall cost of treatment (11–13).

The Brazilian Health Regulatory Agency (ANVISA), the authority responsible for drug regulation in Brazil, has neither approved nor registered glucarpidase for use within its jurisdiction. Conversely, the U.S. Food and Drug Administration (FDA) has authorized its use in patients experiencing methotrexate overexposure with impaired renal function. In contrast, in France, glucarpidase is approved for patients with delayed methotrexate clearance or those at high risk of toxicity, without the prerequisite of acute kidney injury (AKI) (14). Its high cost, along with its limited availability, especially in low-and middle-income countries, underscores the need to explore extracorporeal therapies as a viable and cost-effective alternative.

Extracorporeal therapies have been employed to reduce serum MTX levels due to its molecular properties, although the outcomes have been inconsistent depending on the type of therapy used. Various dialysis modalities have been reported, with variable success rates. While conventional low-flux hemodialysis has proven to be minimally effective in lowering serum MTX concentrations, high-flux hemodialysis has demonstrated efficacy in methotrexate elimination in reported cases. However, well-designed studies are still lacking to comprehensively evaluate the reduction in serum methotrexate levels and the associated rebound effect (15, 16).

In this study, we present a case series of patients with HD-MTX intoxication and multi-organ involvement who were treated with HF-HD, focusing on its relative reduction in serum MTX concentrations. Furthermore, we conducted a literature review of available extracorporeal therapy modalities and their effects on serum MTX concentrations.

## Materials and methods

Between 2018 and 2024, three patients were referred to a nephrologist for HD-MTX nephrotoxicity and required extracorporeal therapies at Hospital de Base de São José do Rio Preto, a university hospital located in São Paulo, Brazil. All data collected for this study were extracted from the electronic health records.

All patients were undergoing their first cycle of chemotherapy and received urinary alkalinization, with MTX administration initiated only after achieving a target urinary pH >7.0. Additionally, they were treated with intravenous fluid expansion and received leucovorin 24 h after the start of the MTX infusion at a rescue dose of 15 mg/m^2^.

Serum MTX levels were measured using the Architect Methotrexate chemiluminescence assay (Abbott Diagnostics, IL, USA) on the Architect 2P49 system (Abbott Diagnostics). The expected MTX levels, measured at specific time points post-administration, associated with toxicity risk are shown in Table 1.

**Table 1:** Expected MTX levels by time.

Time post-administration	MTX levels
24 h	>10,0 μmol/L
48 h	>1.0 μmol/L
72 h	>0.1 μmol/L

The relative reduction in serum MTX concentration was calculated using the pre-dialysis MTX concentration (MTXpre) and post-dialysis concentration (MTXpos) according to the formula: (MTXpre – MTXpos) / MTXpre * 100. Variables were reported as mean and standard deviation or as median and percentiles, where applicable.

## Results

Among the three patients, two were younger than 18 years. All received HD-MTX (3–12 mg/m^2^) administered via a 24-h infusion. Two were diagnosed with hematologic malignancy and the other one with osteosarcoma, as detailed in Table 2.

**Table 2:** Demographic and clinical characteristics of the 3 patients.

	Patient 1	Patient 2	Patient 3
Sex	Male	Female	Male
Age (Years)	16	47	9
Primary diagnosis	B-acute lymphoblastic leukemia	Diffuse large B-cell lymphoma	Osterosarcoma
MTX dosing regimen	3 g/m^2^ during 24 h	3,5 g/m^2^ during 24 h	12 g/m^2^ during 24 h
Weight (kg)	82	77	20
Body surface área (m^2^)	1.89	1.709	0.76
Baseline serum creatinine (mg/dL)	0.61	0.50	0.40
eGFR (mL/min)*	56	116	70

*eGFR: estimated Glomerular Filtration Rate was calculated using the Revised Bedside Schwartz Formula for patients under 18 years- old and the CKD-EPI Creatinine Equation (2021) for those ≥ 18 years.

### Patient 1

A 16-year-old male patient was admitted with a 4-day history of fever. Laboratory investigation revealed pancytopenia, leading to the diagnosis of B-cell acute lymphoblastic leukemia. The patient’s medical history includes congenital heart disease (single ventricle), for which he underwent Fontan and Glenn procedures 10 years ago. He is currently on enalapril and acetylsalicylic acid, with regular follow-up in cardiology and his baseline serum creatinine was 0.61 mg/dL. Treatment was initiated with the GRAALL protocol, and he received methotrexate at a dose of 3 g/m^2^, administered over a 24-h infusion.

Forty-eight hours post-MTX infusion, the patient exhibited a significant decline in renal function, evidenced by an elevated creatinine level of 2.17 mg/dL, alongside exacerbation of pancytopenia. On day six following MTX administration, the patient demonstrated persistent deterioration in hematologic parameters, with methotrexate plasma levels exceeding 1.500 μmol/L, in addition to presenting with mucositis and elevated bilirubin levels (4.7 mg/dL).

Despite the improve in renal function and the rescue therapy with leucovorin and urinary alkalinization, there was no observed MTX concentration decline, and the first HF-HD was performed after 15-days post-MTX administration when serum MTX was 0.670 μmol/L. He underwent five HF-HD sessions until MTX serum concentrations were less than 0.100 μmol/L, by which time an improvement in the blood count was observed. Clinical and laboratory parameters were improved, and patient was discharged from the hospital 33-days after MTX administration.

### Patient 2

A 47-year-old female patient was admitted with a 3-day history of abdominal pain, nausea, and vomiting, occurring seven days after MTX infusion 24-h at a dose of 3.5 g/m^2^, administered as part of the R-CHOP protocol for the treatment of diffuse large B-cell lymphoma of the breast for relapse prophylaxis in the central nervous system. The patient had no prior comorbidities and was not on regular medication. Laboratory investigation revealed AKI (creatinine: 7.9 mg/dL, baseline value: 0.5 mg/dL), and vigorous hydration along with leucovorin administration was initiated. Due to the lack of response to the initial treatment, a nephrology consultation was requested, leading to the initiation of hemodialysis. The serum MTX level was measured, with an initial value of 2,678 μmol/L.

The patient did not present with hepatic toxicity but developed mucositis and myelotoxicity. She underwent three HF-HD sessions until MTX serum concentrations were sufficiently reduced to be effectively neutralized by leucovorin, and his kidney function has been improved. After 14 days patient was discharged from hospital.

### Patient 3

V.H.F.A., a 9-year-old male patient diagnosed with osteosarcoma of the right thigh, received HD-MTX at 12 mg/m^2^, administered over a 24-h continuous infusion. His past medical history was notable for intestinal reconstruction at 2 days of life, tricuspid valve atresia with ventriculo-arterial concordance, Glenn procedure and pulmonary artery ligation at 5 months, and a total cavopulmonary Fontan procedure at 6 years. The patient was maintained on warfarin 5 mg daily.

One day post-infusion, he developed diarrhea, nausea, and vomiting, followed by mental confusion and AKI (serum creatinine: 2.3 mg/dL) the next day. He also exhibited mucositis and febrile neutropenia, necessitating the initiation of broad-spectrum antibiotic therapy.

**Figure 1:**
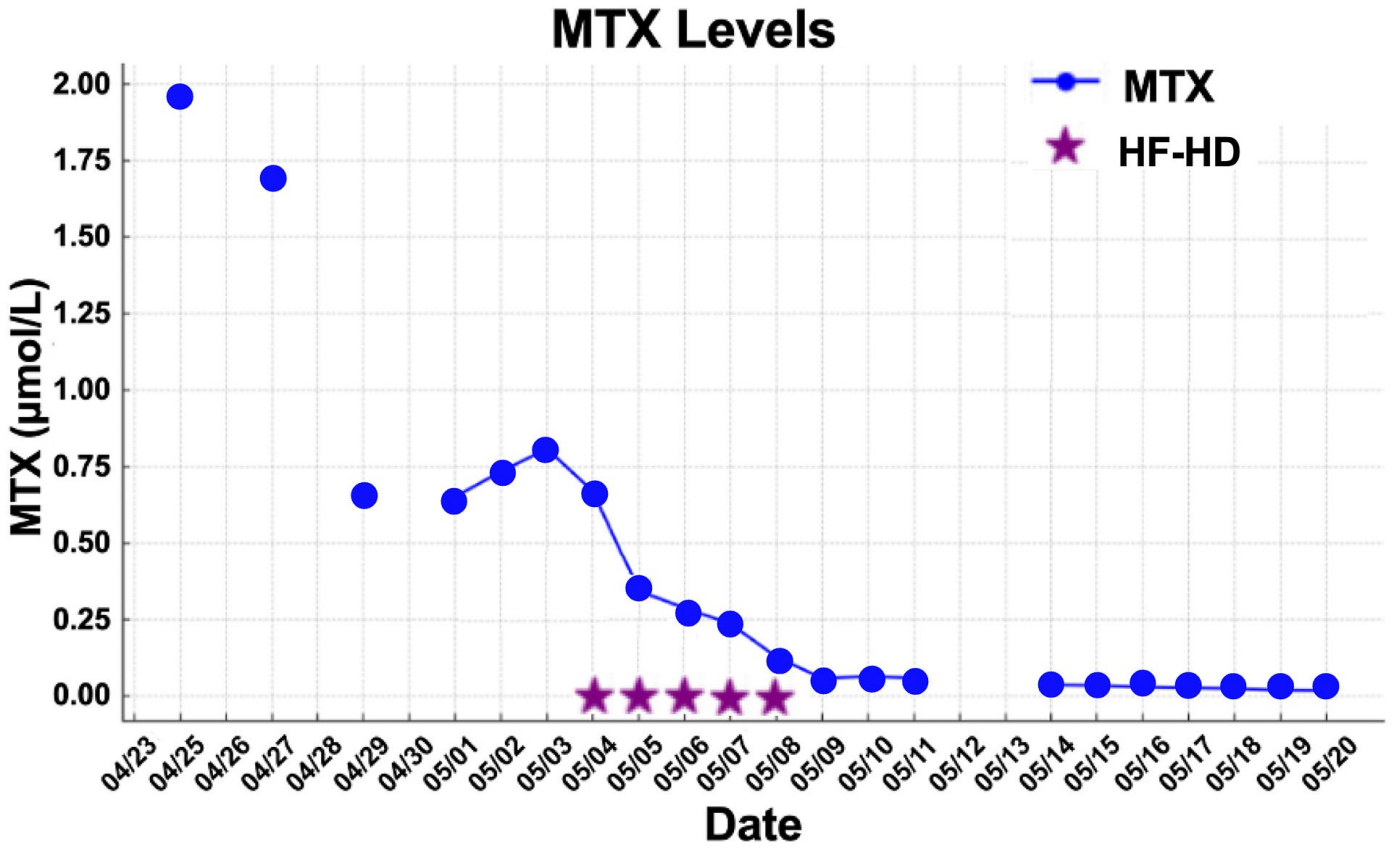
MTX levels vs. date for Patient 1.

MTX levels were markedly elevated, and the patient demonstrated minimal response to conventional rescue therapy, including aggressive hydration and leucovorin, as illustrated in Figure 1. Consequently, the nephrology team was consulted, and renal replacement therapy with HF-HD was initiated. The patient underwent nine sessions of HF-HD, leading to a significant reduction in methotrexate levels and subsequent recovery of renal function. He was discharged from hospital after 42 days.

**Figure 2:**
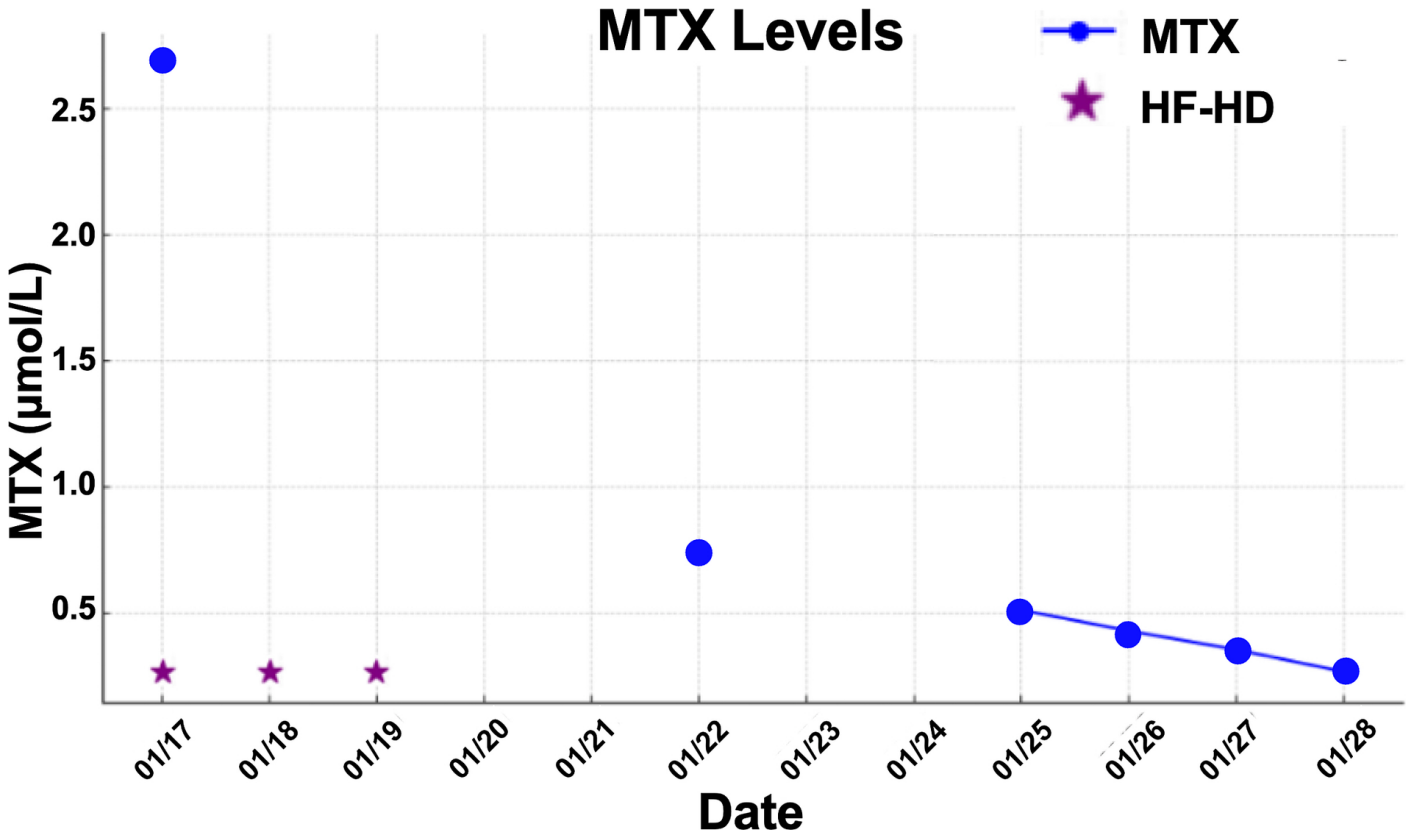
MTX levels vs. date for Patient 2.

**Figure 3:**
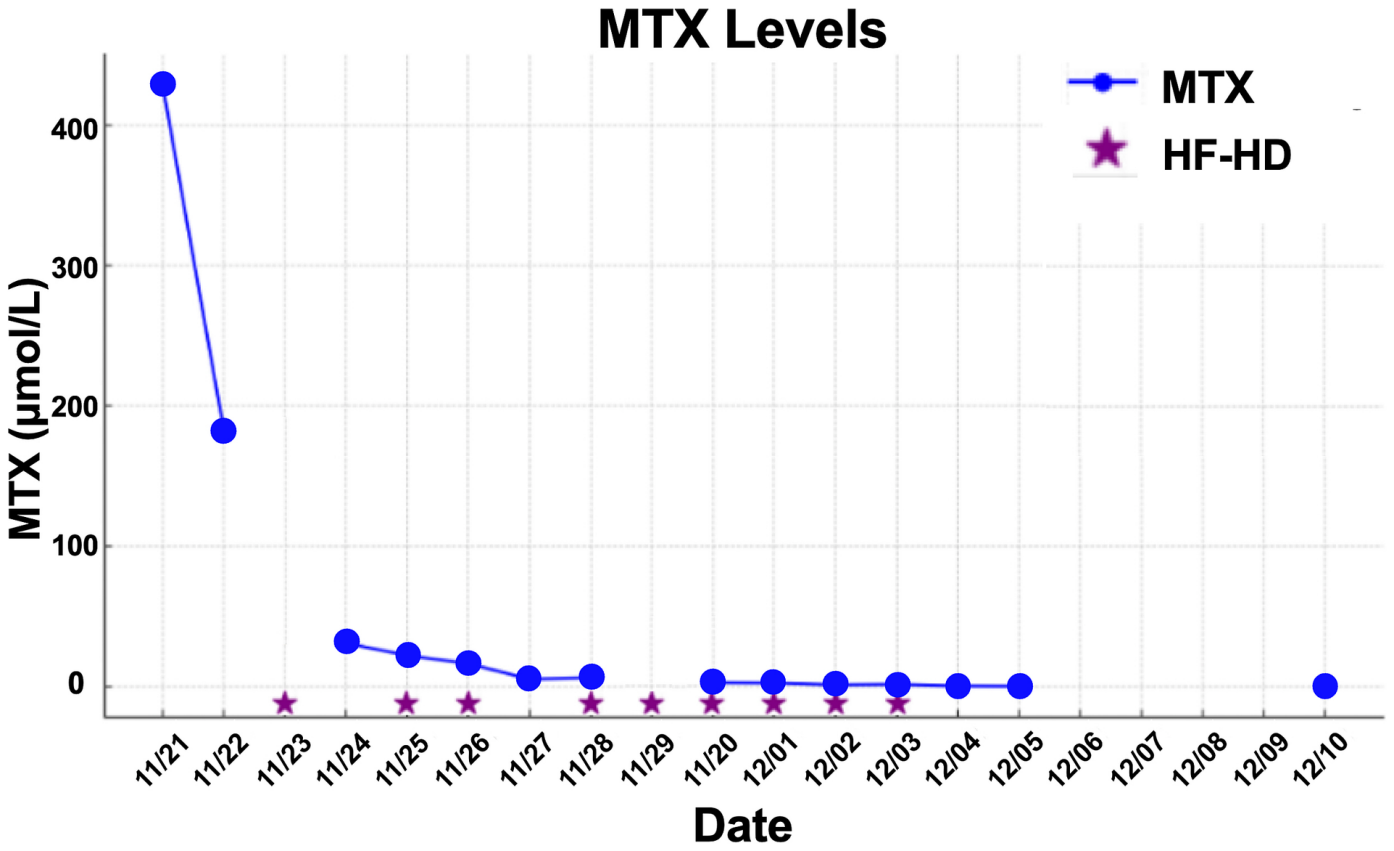
MTX levels vs. date for Patient 3.

The mean number of dialysis sessions required by the patients was 5.3 ± 2.5. All patients were treated using the Fresenius® 4008S V10 machine with a Revaclear 400® dialyzer (UF-Coefficient: 54 mL/h*mmHg and Effective Membrane Area: 1.8 m^2^) or FX60® (UF-Coefficient: 40 mL/h*mmHg and Effective Membrane Area: 1.3 m^2^). The mean relative reduction rate of MTX was 44.5 ± 19.1%. The dialysis characteristics and individual MTX relative reduction rates are detailed in Table 3. As shown in Figures 1–3, not all HF-HD sessions had serum MTX levels measured due to the availability of the test in our institution.

**Table 3:** Dialysis characteristics and effect on serum MTX.

	Patient 1	Patient 2	Patient 3
Number os sessions	5	3	9
Modality	HF-HD	HF-HD	HF-HD
Dialyzer	Revaclear 400®	Revaclear 400®	F60S®
Blood flow rate (mL/min)	200 [200–300]	150 [150–200]	100 ± 0
Dialysate flow rate (mL/min)	300 [300–500]	300 [300–500]	300 ± 0
Session time	4 [2,75–4]	3 ± 0	3 ± 0
Relative reduction MTX (%)	45.4 ± 23	22.5	50.4 ± 10.2

## Discussion

HD-MTX toxicity is acknowledged as an oncologic emergency, given its potential to induce severe organ damage and present a significant life-threatening risk. The administration of leucovorin rescue, coupled with aggressive hydration and urinary alkalinization, has markedly reduced the incidence of MTX toxicity (1). Nevertheless, in cases where this conventional approach proves inadequate, glucarpidase has become the preferred treatment; however, its widespread use remains limited due to its high cost (17). In such scenarios, extracorporeal therapies have been proposed as a viable salvage strategy. In this study, we report three cases of HD-MTX toxicity successfully treated with HF-HD.

Studies indicate that 0.5–1.8% of patients undergoing HD-MTX therapy experience elevated serum levels following infusion, increasing their susceptibility to toxicity (18–20). The most reliable marker of HD-MTX toxicity, as identified in prior research, is delayed clearance, defined by a serum MTX concentration of ≥1 μmol/L (0.454 mg/L) at 48 h post-infusion. However, the incidence and severity of organ-specific toxicity can vary based on study design and the characteristics of the population studied (21, 22). In the present study, all cases exhibited serum MTX levels above this critical threshold.

MTX is a low-molecular-weight compound (454 Da) that exhibits moderate protein binding, typically in the range of 30 to 60%. The volume of distribution is approximately 0.18 L/kg in acute exposure, expanding to 0.4–0.8 L/kg with prolonged administration. While 10–20% of MTX is eliminated via biliary excretion, the majority is cleared renally in its unchanged form, typically within 48 h (23). Renal excretion involves both glomerular filtration and tubular secretion; however, the latter can become saturated under conditions of high-dose MTX administration. Consequently, MTX clearance rates have been documented to vary between 40 and 200 mL/min, depending on serum concentrations (24). These pharmacokinetic attributes underscore the viability of extracorporeal therapies for MTX removal, as corroborated by both *in vitro* investigations and animal studies (25, 26). However, these therapies are frequently associated with the phenomenon of rebound, characterized by a post-procedural increase in serum MTX concentrations, with an average rebound of approximately 76% relative to pre-session levels. This effect is likely attributable to the redistribution of MTX from intracellular compartments and peripheral tissues into the vascular compartment. The magnitude of the rebound is more pronounced with highly efficient modalities, exemplified by intermittent dialysis, which may necessitate additional sessions to ensure sufficient and sustained MTX clearance (27).

The EXTRIP workgroup provides a strong recommendation, albeit based on low-quality evidence, against the use of extracorporeal therapy for MTX intoxication when glucarpidase is available. However, in settings where glucarpidase is not accessible, this recommendation weakens. These guidelines are grounded in the reported risks associated with the procedure, such as hypotension, ventricular tachycardia, myocardial infarction, cardiac arrest, electrolyte imbalances including hypokalemia and hypophosphatemia, and catheter-related bleeding—complications commonly observed with traditional indications for this treatment. Furthermore, extracorporeal therapy primarily removes MTX from the intravascular compartment, with minimal impact on intracellular stores, where the drug exerts its effect. Another significant concern is that these therapies also clear leucovorin, which is crucial for the restoration of the folate cycle (23). All three of our patients received optimized therapy but did not achieve an adequate response after at least 72 h, increasing their risk of complications due to persistently elevated serum MTX levels, which ultimately led the team to initiate extracorporeal therapies.

When extracorporeal therapy is chosen, several modalities have been reported in the literature. Murashima et al. (28) and Aristizabal-Alzate et al. (29) documented the use of peritoneal dialysis (PD) to manage MTX intoxication in patients with chronic kidney disease (CKD). Both cases involved intensified dialysis regimens, which resulted in favorable outcomes. It was observed that the MTX clearance via PD was 0.124 mL/kg/min, accounting for approximately 19% of the total systemic clearance of the drug. PD has also been used in patients with AKI, though no significant reduction in serum MTX levels was reported. Diskin et al. conducted the first study comparing HF-HD and PD in the same patient, showing that MTX clearance was lower with PD than with HF-HD. Although the clearance rates were similar during the first hour, the continuous refreshment of dialysate in HF-HD maintains a high concentration gradient between compartments, facilitating more efficient removal throughout the session. Conversely, MTX serum levels showed minimal change with PD, likely due to the slow fluid exchange characteristic of this modality, resulting in a slower rebound of the free MTX fraction. In contrast, HF-HD reduced MTX serum levels by approximately 25%, with a significant rebound observed within two hours after the session (30). In the acute setting, PD is only recommended when HF-HD is not available (27).

In situations where extracorporeal therapy is required, conventional low-flux hemodialysis has demonstrated inadequate efficacy in the removal of MTX, leading to the adoption of intermittent HF-HD as the preferred modality (23). Wall et al. (31) documented the effectiveness of HF-HD in six patients, half of whom were undergoing maintenance dialysis due to end-stage chronic kidney disease, while the remainder developed AKI during treatment. In this study, detailed measurements of serum and dialysate MTX concentrations in patient 1, who was on chronic dialysis prior to MTX administration, revealed that 63% of the infused MTX was eliminated within six hours of therapy. The plasma clearance rate of MTX, calculated 30 min after therapy initiation, was 118.3 ± 9.5 mL/min based on arterial–venous serum MTX differentials, and 95.4 ± 13.0 mL/min based on dialysate MTX levels. These elevated clearance rates persisted for three hours after the commencement of therapy. Additionally, all patients exhibited a rebound in serum MTX concentrations two hours after cessation of therapy, a finding consistent with our patient 1, who had the most accurate pre-and post-HF-HD serum MTX measurements, as well as post-therapy assessments to quantify the rebound effect.

Although MTX clearance was not measured in all sessions, in our study, the relative reduction rate of MTX was lower than previously reported in the literature (76% [42–94%]), likely due to our use of lower blood flow rates (100 [100–200] mL/min vs. 300–400 mL/min), dialysate flow rates (300 [300–300] mL/min vs. 500–800 mL/min), and shorter therapy durations (3 h vs. 4.4 h) compared to protocols implemented in earlier studies (7).

Sakran et al. examined the half-life of MTX and determined a median of 4.4 h, which closely resembles the values seen in patients with normal renal function (2–4 h). Additionally, they assessed both hemodiafiltration (HDF) and HF-HD in the same patients across different sessions, finding no significant improvement in clearance with the addition of convective clearance. This result is consistent with expectations, given the low molecular weight of MTX (7).

Recent developments have highlighted an increasing interest in the application of advanced extracorporeal therapies. In a case reported by Chan WKY et al., a sequential treatment approach was employed for an 11-year-old patient suffering from MTX intoxication. This approach involved the combination of single-pass albumin dialysis (SPAD) with continuous dialysis equipment and hemoperfusion (HP) using a charcoal filter, and it was found to be both safe and effective in reversing the toxicity (32).

SPAD, a therapy utilizing continuous dialysis systems with albumin-enriched dialysate, has been shown to be effective in treating exogenous poisonings, including those induced by MTX and carbamazepine (33). However, a case report by Vilay et al. (34) involving a 13-year-old patient treated for MTX intoxication through various therapies found no significant difference in MTX clearance when albumin was excluded from the dialysate. Additionally, the high cost of SPAD remains a limiting factor in its widespread use.

Advanced extracorporeal therapies, including SPAD (single-pass albumin dialysis), Molecular Adsorbent Recirculating System (MARS), and therapeutic plasma exchange (TPE), have been identified as viable strategies for addressing severe cases of methotrexate (MTX) toxicity. These modalities are particularly indicated in instances of multi-organ failure, such as the concurrent presentation of acute kidney injury (AKI) and acute liver failure, as documented in the literature. Corbisier et al. (27) provide a comprehensive evaluation of these therapies, underscoring their critical role in scenarios where conventional extracorporeal approaches prove inadequate.

Charcoal HP has proven to be particularly adept at eliminating both hydrophilic and lipophilic toxins. Although initially developed for exogenous intoxications, the use of charcoal filters has diminished with advancements in capillary and dialyzer technology. Additionally, this therapy does not address fluid or electrolyte imbalances, which can be problematic in patients with renal dysfunction. Another limitation is its high cost, as charcoal cartridges typically become saturated within 3–4 h, necessitating frequent replacements (35, 36).

High cut-off (HCO) membrane hemodialysis has been reported as a potential approach for treating HD-MTX intoxication in the context of AKI. Studies have shown that this method achieves approximately a 50% reduction in MTX levels during dialysis, with the expected post-dialysis rebound in concentrations. However, despite its effectiveness, HCO dialysis tends to be more expensive when compared to HF-HD (37).

This study has several limitations that should be acknowledged when interpreting the results. First, the sample size is limited to only three patients, reflecting the low number of HD-MTX toxicity cases and the constrained scope of the study setting. The heterogeneity among patients, including differences in age, underlying conditions, and HD-MTX dosages, further limits the generalizability of the findings.

Additionally, there were gaps in data collection. Pre-and post-dialysis methotrexate concentrations and clearance rates were not consistently measured in all HF-HD sessions due to resource limitations at the hospital. These data deficiencies may impact the accuracy of efficacy evaluations and represent a barrier to more comprehensive analyses. Furthermore, the absence of a control group, such as patients treated with glucarpidase or other extracorporeal therapies, is a notable limitation. Although the unavailability of glucarpidase is mentioned, it is essential to emphasize that this restricts direct comparisons. To mitigate this limitation, we have indirectly compared our findings with data available in the literature.

We acknowledge that these limitations constrain the extrapolation of the results; however, we believe this study provides valuable insights into the management of HD-MTX toxicity and the use of HF-HD, particularly in resource-limited settings where alternative therapeutic options may be scarce. By documenting real-world applications and outcomes, it underscores the importance of optimizing extracorporeal strategies to enhance patient care in high-stakes oncologic emergencies.

In conclusion, for patients experiencing HD-MTX intoxication, when glucarpidase is unavailable, HF-HD presents an effective treatment option, with a lower cost compared to other extracorporeal therapies. Serum MTX rebound occurs, and repeated sessions may be necessary to ensure adequate patient management.
